# MicroRNAs from extracellular vesicles as a signature for Parkinson's disease

**DOI:** 10.1002/ctm2.357

**Published:** 2021-04-05

**Authors:** Lucas Caldi Gomes, Anna‐Elisa Roser, Gaurav Jain, Tonatiuh Pena Centeno, Fabian Maass, Lukas Schilde, Caroline May, Anja Schneider, Mathias Bähr, Katrin Marcus, André Fischer, Paul Lingor

**Affiliations:** ^1^ Department of Neurology School of Medicine Klinikum rechts der Isar Technical University of Munich München Germany; ^2^ Department of Neurology University Medical Center Göttingen Göttingen Germany; ^3^ Department for Epigenetics and Systems Medicine in Neurodegenerative Diseases German Center for Neurodegenerative Diseases (DZNE) Göttingen Germany; ^4^ Medical Faculty Medizinisches Proteom‐Center Ruhr University Bochum Bochum Germany; ^5^ Medical Proteome Analysis Center for Protein Diagnostics (PRODI) Ruhr‐University Bochum Bochum Germany; ^6^ Department of Neurodegenerative Diseases and Geriatric Psychiatry University Clinic Bonn and German Center for Neurodegenerative Diseases (DZNE) Bonn Germany; ^7^ Department of Psychiatry and Psychotherapy University Medical Center Göttingen Göttingen Germany; ^8^ German Center for Neurodegenerative Diseases (DZNE), site München München Germany

Dear Editor,

In the present study, we have demonstrated that extracellular vesicles (EVs) derived from cerebrospinal fluid (CSF) represent a promising source for the identification of a novel miRNA signatures in Parkinson's disease (PD). Using next‐generation small‐RNA sequencing, we present for the first time the complete and quantitative microRNAome of EVs isolated from human CSF of PD and age‐correlated controls (CTR). In parallel, we performed CSF proteomic profiling of overlapping patient cohorts, which revealed the deregulation of disease‐relevant pathways similar to the ones obtained with the parallel miRNA analyses, supporting the results for the identified signature.

Novel molecular signatures and disease biomarkers are urgently needed for PD, not only to improve diagnostic precision, but also to enable monitoring of treatment responses, as well as stratification of patients according to the molecular background, rather than solely on clinical phenotypes.^1^ Circulating miRNAs are auspicious targets for biomarker studies because their expression reflects the functional state of cells and is directly influenced by pathological stimuli.^2^ CSF is in direct contact with the brain parenchyma, and molecular alterations in its composition may reflect specific changes related to PD pathology in the brain.

MiRNA species circulating in CSF seem to overlap with miRNAs expressed in brain tissue.^3^ Furthermore, miRNAs and other small‐RNAs are enriched in the vesicular fraction of human CSF.^3^, ^4^ In order to characterize the size and particle distribution in our CSF EV preparations, we used Nanoparticle Tracking Analyses and observed a similar enrichment as previously reported (Figure 1A). Small‐RNA sequencing ratified the miRNA abundance in CSF EVs—they represented, on average, 97.4% of all mapped small‐RNAs in the discovery cohort (Figure 1C). In total, we detected 688 miRNAs. A total of 208 of these had a base mean higher than 5 reads and were analyzed further. Differential expression analyses revealed differences in the levels of 22 miRNAs in the PD versus CTR comparison (Figure 1D). The majority of the differentially expressed miRNAs were upregulated in PD subjects, whereas downregulated miRNAs showed only subtle levels of deregulation (–0.60 ≤ log_2_FC ≤ –0.16). Among upregulated species figured brain‐enriched miRNAs miR‐9‐5p, let‐7b, miR‐181a‐5p, and miR‐181b‐5p (Figures 1D and 1E). To reduce bias by a potential erythrocyte contamination, we strictly selected CSF samples with a low number of red blood cells (<100/μl CSF). Furthermore, because miR‐451a is highly enriched in red blood cells, it was excluded from feature‐selection analyses.

**FIGURE 1:**
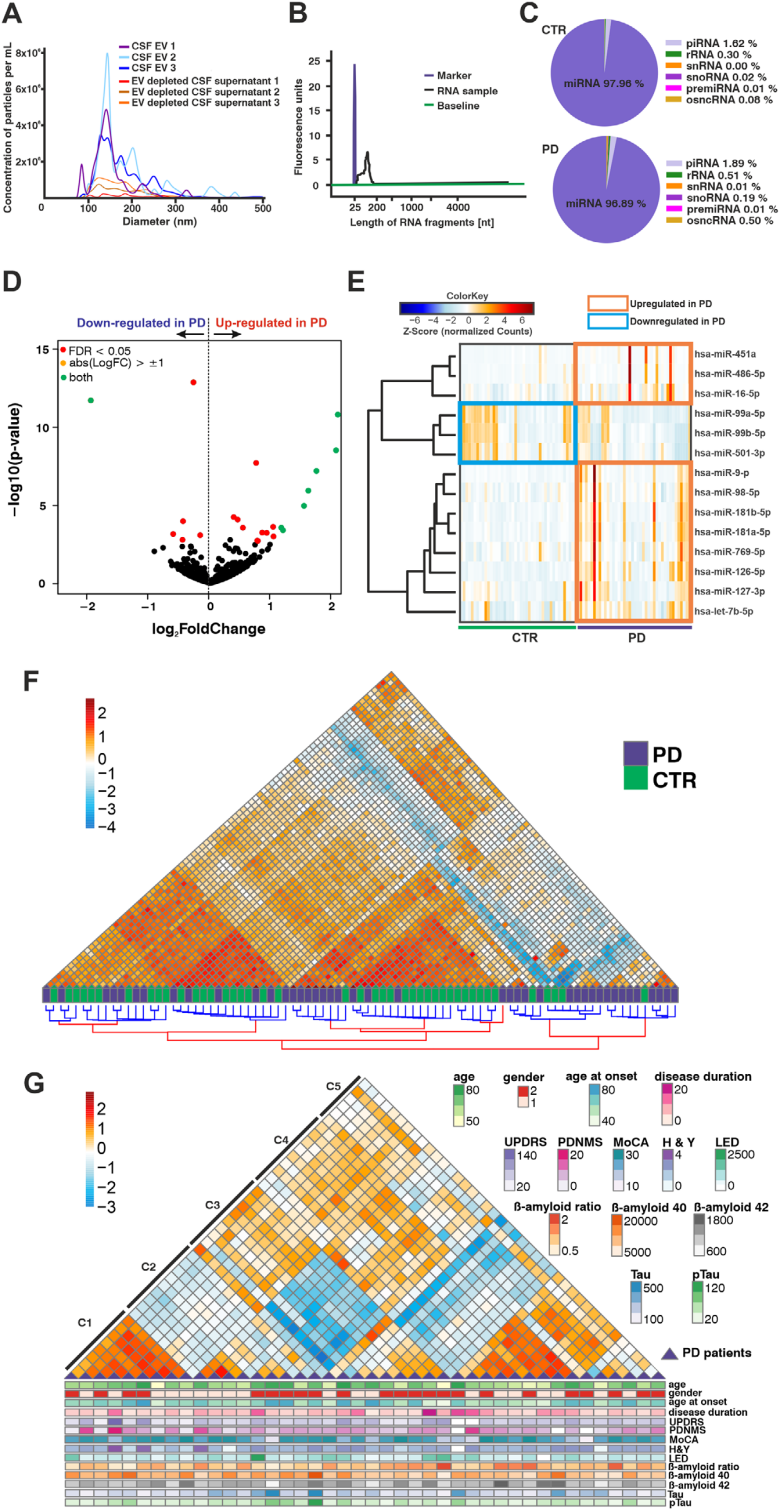
Small RNA sequencing from CSF EVs. (A) Size and particle distribution in EV‐enriched CSF and EV‐depleted supernatant fractions obtained with nanoparticle tracking analysis (NTA) after CSF ultracentrifugation. (B) Electropherogram showing the profile of RNA isolated from CSF extracellular vesicles. (C) Pie charts showing the average proportions of the different small RNA species in the small RNA libraries of CTR and PD subjects. (D) Volcano plot showing all detected miRNAs in the CSF of PD patients and CTR. Significantly different expressed miRNAs between CTR and PD are indicated in red (FDR < 0.05) and green (FDR < 0.05; log_2_FC > 1). (E) Heatmap with individual expression levels for significantly regulated miRNAs in the discovery cohort (PD, *n* = 42; CTR, *n* = 43). (F) Hierarchical clustering of PD patients and CTR subjects in the discovery cohort. Samples with similar miRNA expression profiles are located close to each other. (G) Unbiased hierarchical clustering of PD patients from the discovery cohort according to similar miRNA expression profiles, showing also the corresponding clinical parameters. CTR, control; PD, Parkinson's disease; CSF, cerebrospinal fluid; EVs, extracellular vesicles; NTA, nanoparticle tracking analysis; C1–C5, distinct PD sample clusters; sex: 1 = female, 2 = male; age, age at onset and disease duration given in years; UPDRS, Movement Disorders Society‐Unified Parkinson's Disease Rating Scale; PDNMS, Parkinson's Disease Non‐Motor Scale; MoCA, Montreal Cognitive Assessment; H&Y, modified Hoehn & Yahr stage; LED, levodopa equivalent dose in mg; beta‐amyloid‐(1‐40) and beta‐amyloid‐(1‐42) in pg/mL; beta‐amyloid ratio: beta‐amyloid‐(1‐42)/beta‐amyloid‐(1‐40) × 10; Tau in pg/mL; pTau: phosphorylated (181) Tau in pg/mL

To explore the overall miRNA expression differences in the cohorts, hierarchical clustering analyses were performed (Figure 1F). Grouping samples based on miRNA expression levels revealed differences in the overall miRNA abundance between PD and CTR samples. PD samples showed expression heterogeneity, as some of these subjects clustered close to/among CTRs. Repeating the analysis with PD samples only revealed five different subclusters (Figure 1G) that did not correlate with the distribution of clinical parameters (e.g., disease duration; age of death; Levodopa‐equivalent dose; scores for disease severity [PDNMS; MDS‐UPDRS; MoCA; mH&Y]). This suggests a molecular diversity in PD cases that is reflected by miRNA expression.

Using machine learning approaches (measure of relevance [MoR]; reliability analysis [RiA]; random forest) with the small‐RNA sequencing data, we found an iterative signature comprising miR‐126‐5p, miR‐99a‐5p, and miR‐501‐3p, which could differentiate PD and CTR samples in our discovery cohorts (42 PD; 43 CTR) (Figures 2A–2D). Sample numbers for the discovery cohort were similar to other studies in the field^4^ and were shown to be adequate for algorithm training. The panel was able to classify PD/CTR samples in an independent validation cohort (nine PD; 11 CTR) with an area under the curve (AUC) value of 0.85 (Precision–recall AUC = 0.88; sensitivity = 0.78; specificity = 0.95). Mean decrease in Gini (Figure 2D) indicated miR‐126‐5p as the most discriminative variable, followed by miR‐99a‐5p and miR‐501‐3p. A third independent cohort (25 PD; 25 CTR) was used for validation purposes. Real‐Time Quantitative Reverse Transcription PCR (qRT‐PCR) experiments confirmed the differential expression of miR‐126‐5p and miR‐99a‐5p when comparing PD and CTR cohorts (Figure S2). The individual expression of each signature miRNA in PD subjects of the discovery cohort (Figure 2E) delineated a similar heterogeneity to the one observed in the global miRNA analysis (Figure 1G), confirming the molecular diversity within PD cases. Subclusters 1 and 3 present opposing expression for the signature miRNAs, whereas subclusters 2 and 5 present similar levels for these candidates. These findings suggest that the identified signature would be a useful tool for distinguishing disease subgroups based on miRNA expression. On the other hand, the inclusion of additional patients/cohorts with variate compositions might explain the lack of reproducibility of studies in the field,^5^ as well as the discrepant results for some candidates during the additional validation studies we presented here. Using samples from patients with different molecular backgrounds, which cannot be distinguished by clinical phenotype alone, as well as the smaller size of the validation cohort might explain the differences in the results observed for miR‐501‐3p with RNA sequencing and qRT‐PCR experiments.

**FIGURE 2:**
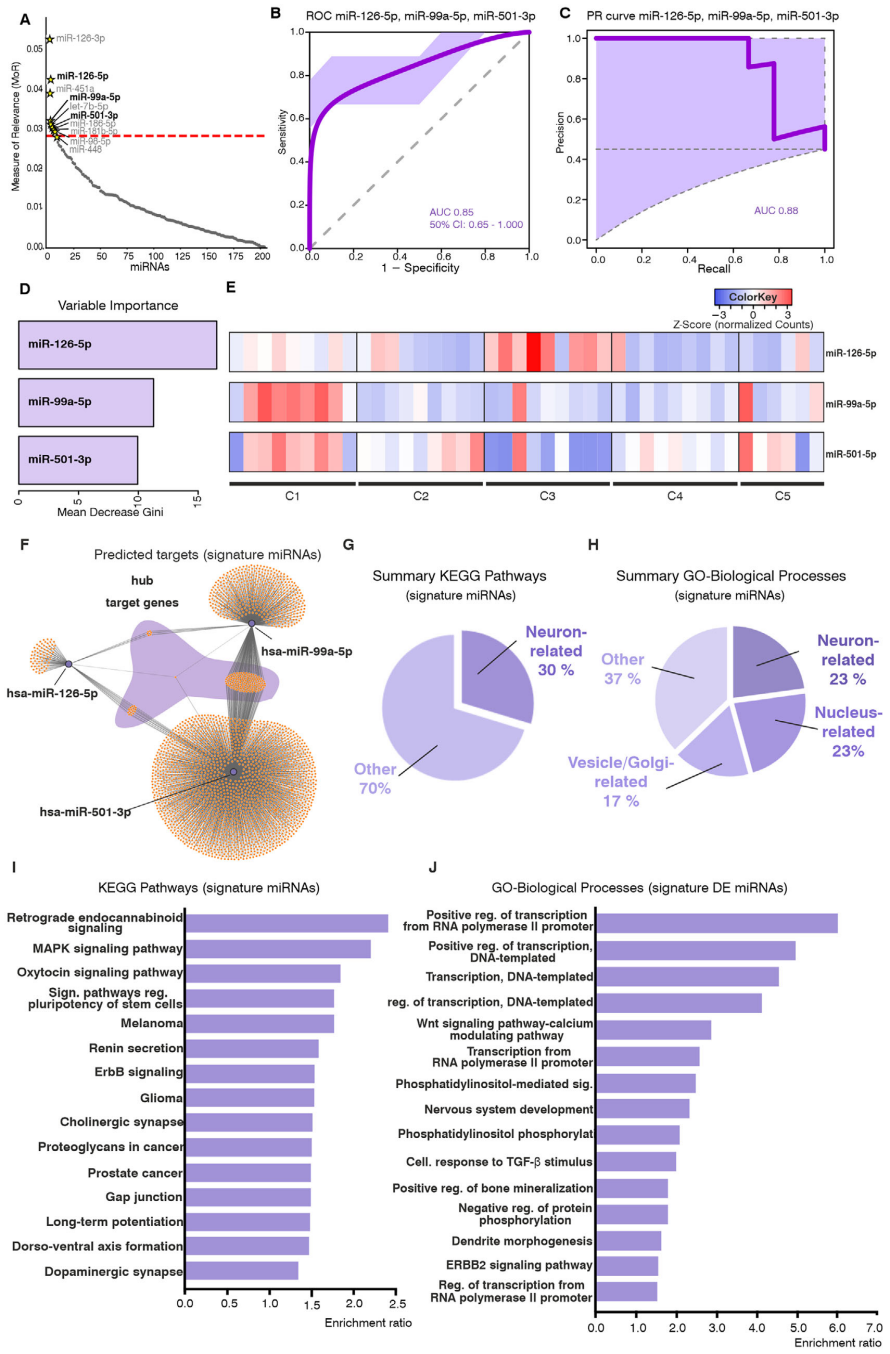
A miRNA signature identified in PD CSF. (A) MoR analysis for identification of relevant miRNAs that discriminate between PD and CTR in the discovery cohort (PD, *n* = 42; CTR, *n* = 43). The red line indicates the critical MoR value cutoff. miRNAs located above this line are considered relevant. The light‐gray miRNAs were excluded after mean score filtering in the feature selection procedure. The combination of miRNAs that was tested to discriminate PD and CTR subjects in an independent validation cohort is indicated in bold black lettering (miR‐126‐5p, miR‐99a‐5p, and miR‐501‐3p). (B) ROC curve showing the performance of the three signature miRNAs for the discrimination of PD and CTR subjects in an independent validation cohort (PD, *n* = 9; CTR, *n* = 11). Training of the model was performed on the discovery cohort with a 10‐fold cross‐validation. The lilac area indicates the 50% confidence interval; an AUC of 0.85 was obtained. (C) Precision–recall curve for the performance of the three signature miRNAs in the validation cohort with an AUC of 0.88. (D) Variable importance indicated by the mean decrease in Gini showing the relevance of the individual miRNAs in the signature. (E) Heatmap showing individual expression levels for the three signature miRNAs in the distinct PD sub‐clusters from the discovery cohort, identified by unbiased hierarchical clustering (PD, *n* = 42). (F–J) Differential expression and target‐gene functional annotation results for CSF small RNA sequencing. (F) Predicted targets for signature miRNAs. Hub target genes common to the three miRNAs are highlighted by the shadowing in purple. (G) Summary of enriched KEGG pathways and (H) GO*‐cellular compartment* categories in the functional annotation for predicted targets of the signature miRNAs. Neuron‐related pathways are enriched in the dataset. (I) Top‐15 KEGG pathways and (J) GO*‐biological processes* terms enriched for the predicted targets of the signature miRNAs. Bars represent the enrichment ratio results from the WEBGESTALT algorithm. CTR, control; PD, Parkinson's disease; MoR, measure of relevance; ROC, receiver operatin characteristic; AUC, area under the curve; C1–C5, distinct PD sample clusters; CSF, cerebrospinal fluid; GO, gene ontology; KEGG, Kyoto Encyclopedia of Genes and Genomes; FDR, false discovery rate

Regarding the biological role of the three signature miRNAs, functional annotation analyses with their predicted targets indicated that these candidates likely originate in neurons. Neuron‐related terms comprised the most frequent enriched categories for *Gene Ontology‐Biological Processes (GO‐BP)* results (8/35 enriched GO‐BP terms), indicating their neuronal origin. Terms including *neuron death*, *vesicle‐mediated transport*, and *proteasomal‐protein catabolic process* indicate the participation of these miRNAs in processes directly related to PD pathogenesis^6^ (Figures 2H and 2J). These findings are corroborated by KEGG pathway (*Kyoto Encyclopedia of Genes and Genomes*) enrichment results: 19 out of 64 annotated KEGG pathways were neuron related (Figures 2G and 2I). Among the top 15 pathways figure *retrograde endocannabinoid signaling* and *cholinergic‐dopaminergic synapse*, categories with important involvement in PD pathology.^6^, ^7^ Furthermore, each candidate of our panel has been linked to neurodegenerative mechanisms previously: miR‐126 has been linked to insulin/IGF‐1/PI3K signaling and found in increased levels in PD substantia nigra^8^; miR‐99a‐5p has been associated with neuroinflammation/neurodegeneration processes by regulating microglial functions^9^; miR‐501‐3p is a regulator of dendritic spine remodeling, and was also found upregulated in Alzheimer's disease brains.^10^

Aiming to identify differentially expressed proteins and to explore disease‐relevant pathways further, an overlapping cohort (64 PD; 61 CTR) was analyzed using mass spectrometry using total CSF (Figure 3A). In total, 67 proteins were found differentially expressed between conditions (45 downregulated in PD/22 upregulated in PD) (Figure 3B). Functional annotation showed an important enrichment for inflammatory/immune‐related terms, as well as neuronal‐related terms (e.g., *axon regeneration*; *neuronal development*; *synapse organization* for GO‐BP terms; *complement/coagulation cascades* for KEGG pathways) (Figure 3D). Remarkably, these results overlap with the pathways annotated for the signature miRNAs, especially for the regulation of *neuron development*/*morphogenesis* and *synapse‐* and *secretion‐*related terms. PPI networks with deregulated proteins revealed important hub‐proteins (TGOLN2; SCG2; KNG1; APOA4) (Figure 3E). Proteins that have been previously postulated as PD biomarkers (VGF and EPHA4) were also identified in our studies (Table S4). Overall, although the parallel studies differed regarding the analyzed CSF compartments and the cohorts did not overlap completely, several disease‐relevant pathways were coincidental, further supporting the results of the miRNA study.

**FIGURE 3:**
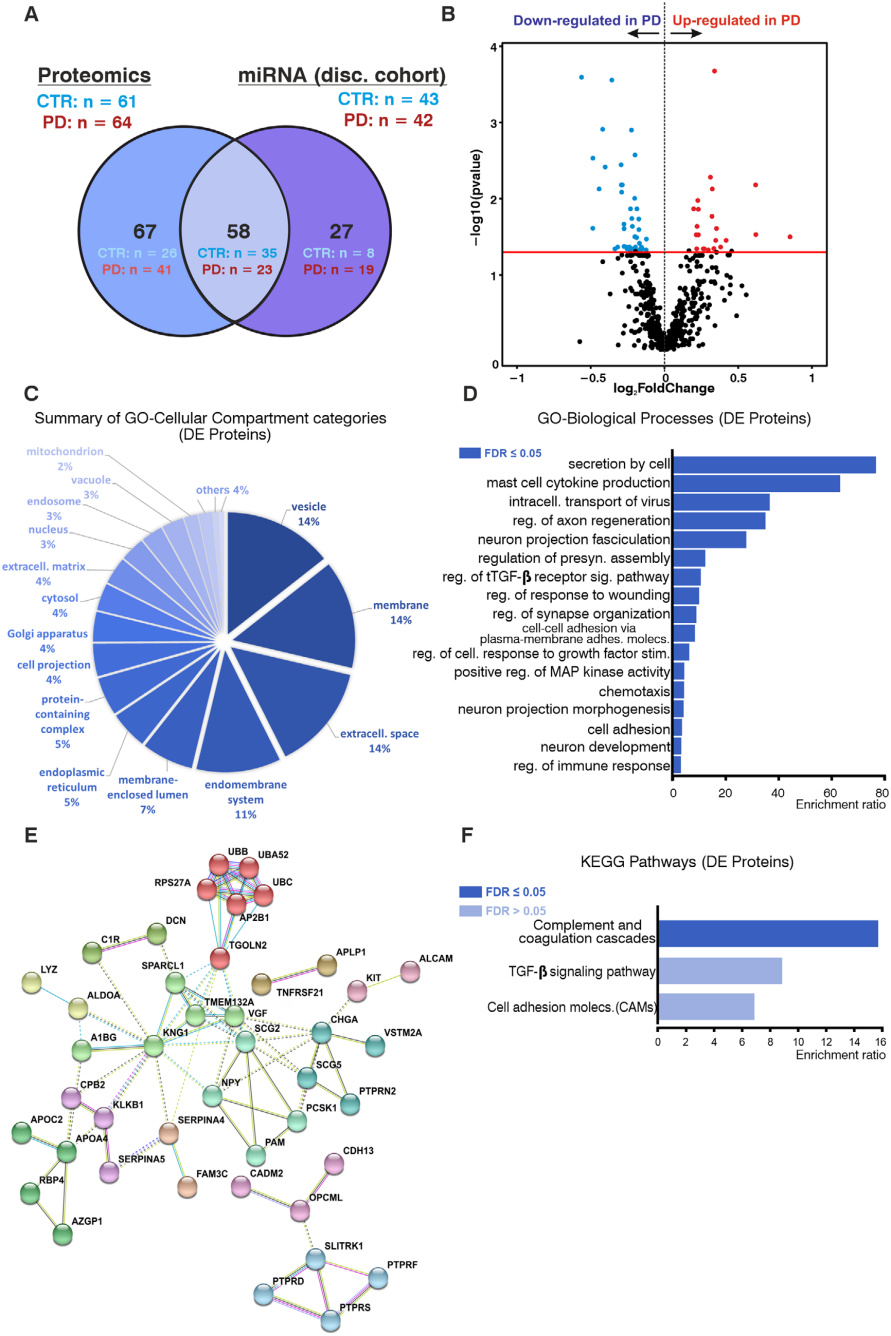
Proteomics analysis with CSF of PD and CTR subjects. (A) Venn diagram indicating the intersection between the discovery cohort from the small RNA sequencing (miRNA) studies and the subgroup of subjects analyzed by proteomics. Stratification of subjects: discovery cohort small RNA sequencing: PD, *n* = 42; CTR, *n* = 43. Proteomics: PD, *n* = 64; CTR, *n* = 61. (B) Volcano plot showing all detected proteins in the total CSF of PD patients and CTR. Differentially expressed proteins between CTR and PD are indicated in blue (downregulated in PD) and red (upregulated in PD) (FDR < 0.05). (C) Summary of enriched GO*‐cellular compartment* categories in the functional annotation for differentially expressed proteins. (D) Enriched GO*‐biological process categories* and (E) KEGG pathway*s* in the functional annotation of differentially expressed proteins. Bars represent enrichment ratio results from the WEBGESTALT algorithm. (F) STRING analysis for the differentially expressed proteins. Clusters defined by the Markov Cluster Algorithm in STRING using default parameters. CSF, cerebrospinal fluid; PD, Parkinson disease; CTR, control subjects; GO, Gene Ontology; KEGG, Kyoto Encyclopedia of Genes and Genomes

Limitations for the identification of molecular signatures in CSF EVs must be critically considered: the starting volume of CSF for isolation of sequencing‐quality RNA (∼4.5 mL) is relatively high, limiting the number of available samples/additional analyses that can be performed. More efficient EV/RNA isolation protocols will significantly improve further CSF multiomics studies. It is important to highlight that the identification of such a miRNA signature in PD CSF must be taken as a starting point, and both the individual expression of each miRNA candidate as well as the combinatorial diagnostic value of the proposed panel must be validated in subsequent multicentric studies. Furthermore, we aimed to strictly select PD patients with a clear clinical phenotype to evaluate miRNA changes in a more advanced stage of the disease. A subsequent study recruiting patients shortly after onset of motor symptoms would be an important follow‐up for this work to assess the value of the signature for the identification of early PD patients.

In summary, we identified a novel miRNA signature in PD CSF composed of miR‐126‐5p, miR‐99a‐5p, and miR‐501‐3p. This signature could potentially contribute to an improved PD diagnosis, as well as to delineate future druggable targets for the disease by revealing important pathophysiological mechanisms. The validity of this signature as a diagnostic biomarker panel should be subsequently validated in larger multicentric studies. Our small‐RNA data also indicate that profiling miRNA expression in CSF EVs might identify clinically inapparent subgroups of PD patients, which could be ultimately used for personalized diagnostic and therapeutic strategies for the disease.

## CONFLICT OF INTEREST

The authors declare no conflict of interest.

## DATA AVAILABILITY STATEMENT

RNA sequencing and proteomics datasets were deposited in public repositories — the European Genome‐phenome Archive (EGA) and the Proteomics Identifications Database (PRIDE), respectively — following the applicable guidelines. RNA sequencing data are stored under the accession number EGAD00001006629. Proteomics data are available via ProteomeXchange with identifier PXD022234.

## Supporting information

Supporting information

Supporting information
