# Supplementary material for: MicroRNAs from extracellular vesicles as a signature for Parkinson's disease
Source: Clin Transl Med. 2021 Apr 5;11(4):e357. doi: 10.1002/ctm2.357 (PMC8021010; doi:10.1002/ctm2.357)
Supplement: Supplementary file 1 — Supporting information [file CTM2-11-e357-s001.docx]

SUPPORTING INFORMATION FOR

**MicroRNAs from extracellular vesicles as a signature for Parkinson’s disease**

Lucas Caldi Gomes*, Anna-Elisa Roser*, Gaurav Jain, Tonatiuh Pena-Centeno, Fabian Maass, Lukas Schilde, Caroline May, Anja Schneider, Mathias Bähr, Katrin Marcus, André Fischer, Paul Lingor

**SUPPLEMENTAL FIGURES**


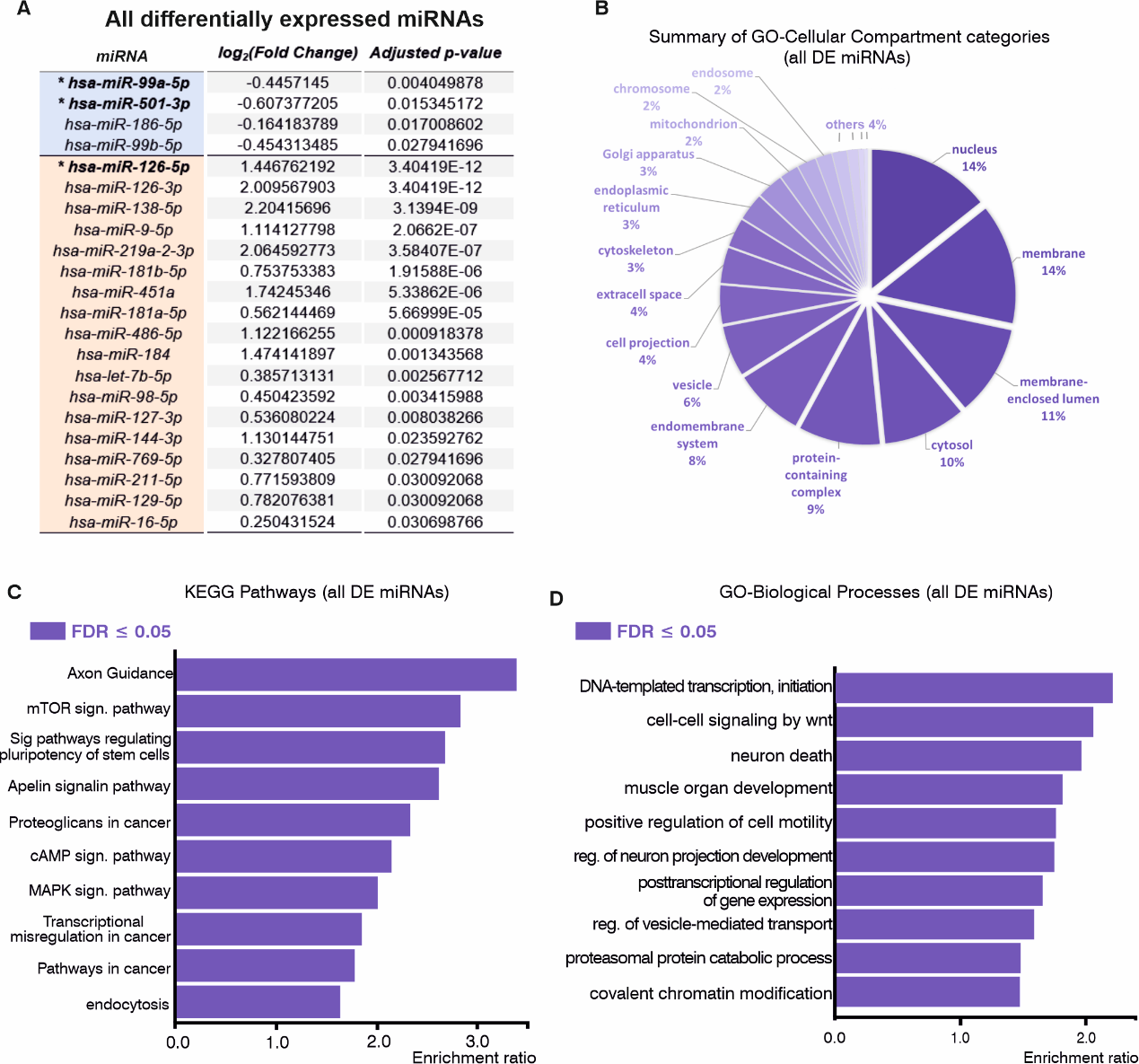


**Fig. S1** Differential expression and target-gene functional annotation results for CSF small RNA sequencing (for all differentially expressed miRNAs). **A** All differentially expressed miRNAs from small RNA sequencing experiments. **B** Summary of enriched GO‒*cellular compartment* categories in the functional annotation for predicted targets of the differentially expressed miRNAs. **C** Top-10 KEGG pathways and **D** GO‒*biological processes* terms enriched for the predicted targets of the differentially expressed miRNAs. Bars represent enrichment ratio results from the WEBGESTALT algorithm.

CSF: cerebrospinal fluid; GO: gene ontology; KEGG: Kyoto Encyclopedia of Genes and Genomes; FDR: false discovery rate

**
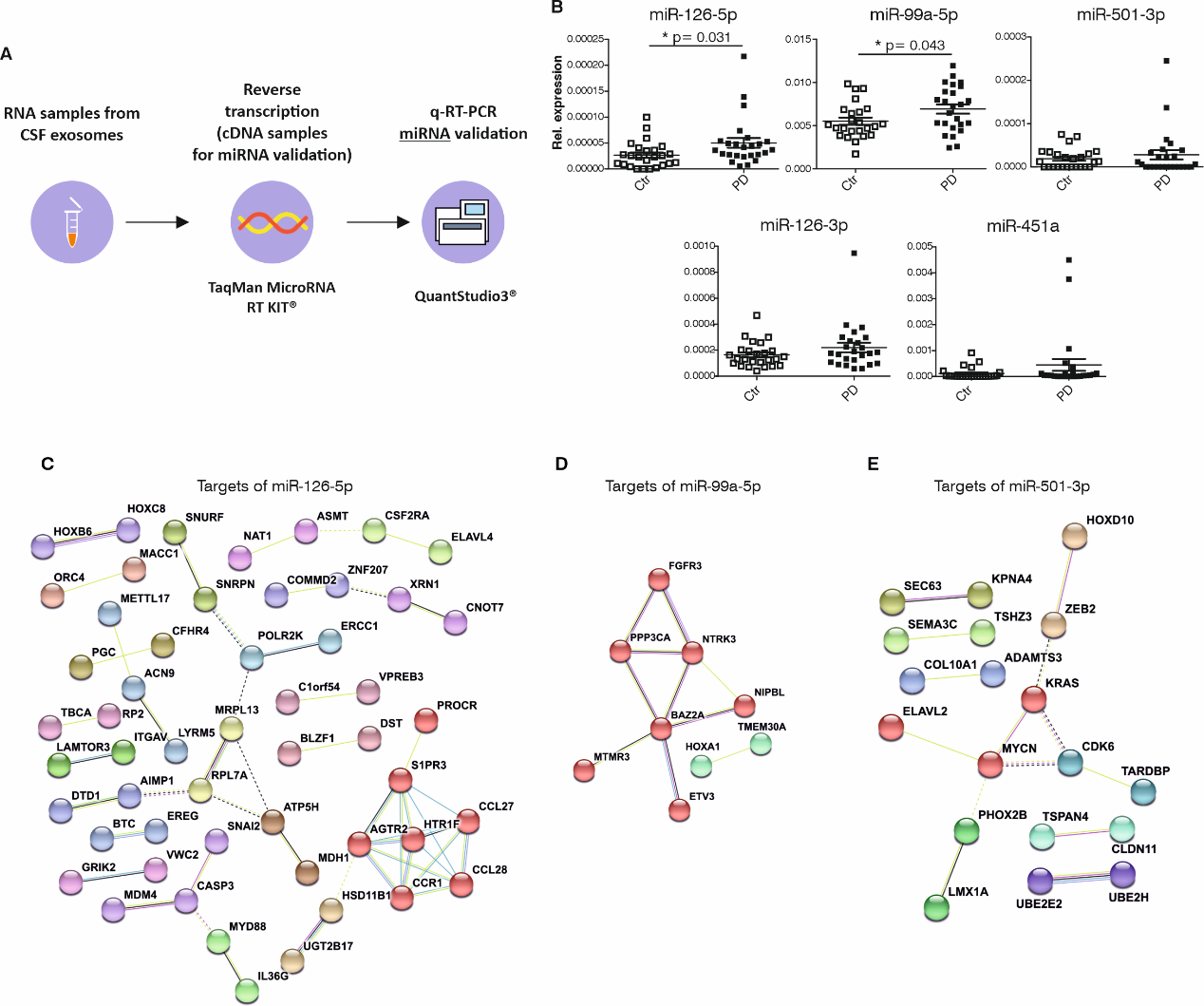
**

**Fig. S2** Validation of sequencing results by q-RT-PCR and STRING analysis for signature miRNAs. **A** Experimental design for validation experiments. **B** Relative expression levels of the 5 most relevant miRNAs identified in the discovery cohort in an independent q-RT-PCR-validation cohort (PD, n = 25; CTR, n = 25). Error bars showing SEM. Data were analyzed by unpaired *t*-test; * p < 0.05. **C‒E** Protein-protein-interaction analyses using the STRING database for the validated targets of miR-126-5p (**C**), miR-99a-5p (**D**), and miR-501-3p (**E**).

PD: Parkinson disease; CTR: control subjects; q-RT-PCR: real-time quantitative polymerase chain reaction.

**SUPPLEMENTAL TABLES**

**Supplemental Table S1:** **Demographic and clinical characteristics of the 3 patient cohorts involved in miRNA analysis.**

|  | **small RNA sequencing discovery cohort**  **(*cohort 1*)** | | **small RNA sequencing validation cohort**  **(*cohort 2*)** | | **qPCR**  **validation cohort**  **(*cohort 3*)** | |
| --- | --- | --- | --- | --- | --- | --- |
|  | **CTR** | **PD** | **CTR** | **PD** | **CTR** | **PD** |
| **Number of patients** | 43 | 42 | 11 | 9 | 25 | 25 |
| **Sex (m/f)** | 23/20 | 23/19 | 8/3 | 6/3 | 15/10 | 15/10 |
| **Age at lumbar puncture (years)** | 65.7 ± 1.8 [37–86] | 66.6 ± 1.8  [44–86] | 70.1 ± 3.5 [51–91] | 67.7 ± 2.3  [60–77] | 68.3 ± 2.4 [41–84] | 67.5 ± 2.3  [39–83] |
| **Disease duration (years)** | NA | 6.4 ± 0.8  [<1–23]  n = 42 | NA | 6.1 ± 1.6  [<1–15]  n = 9 | NA | 4.0 ± 0.8  [<1–16]  n = 23 |
| **MDS-UPDRS total** | NA | 55.1 ± 3.9  [13–144]  n = 40 | NA | 52.8 ± 8.2  [28–94]  n = 5 | NA | 44.9 ± 4.4  [19–111]  n = 24 |
| **mH&Y** | NA | 2.3 ± 0.1  [0–4.5]  n = 41 | NA | 2.9 ± 0.2  [2–4]  n = 9 | NA | 2.1 ± 0.2  [1–3.5]  n = 24 |
| **PD-NMS** | NA | 9.2 ± 0.8  [0–24]  n = 40 | NA | 4.8 ± 1.6  [0–13]  n = 8 | NA | 9.7 ± 1.0  [2–24]  n = 24 |
| **MoCA** | NA | 24.6 ± 0.8  [10–30]  n = 42 | NA | 23.3 ± 1.9  [10–30]  n = 9 | NA | 24.8 ± 0.9  [10–29]  n = 22 |
| **LED (mg)** | NA | 697.9 ± 78.9  [0–2506]  n = 41 | NA | 656.8 ± 239.7  [0–1780]  n = 8 | NA | 474.2 ± 82.8  [0–1375]  n = 24 |

Data are presented as mean ± SEM, and values in squared brackets represent the range. CTR, controls; PD, Parkinson’s disease; MDS-UPDRS, Movement Disorders Society-Unified Parkinson’s Disease Rating Scale; mH&Y: modified Hoehn & Yahr stage; LED: Levodopa equivalent dose. n: number of subjects with available data. Differences between cohorts and between the CTR and PD groups within each cohort were not significant.

**Supplemental Table S2:** **Demographic and clinical characteristics of the cohort used for proteomics experiments (*cohort 4*)**

|  | **CTR** | **PD** |
| --- | --- | --- |
| **Number of patients (overlap with cohort 1)** | 61 (35) | 64 (23) |
| **Sex (m/f)** | 34/27 | 44/20 |
| **Age at lumbar puncture (years)** | 64.9 ± 1.7  [37–92] | 66.5 ± 1.7  [38–86] |
| **Disease duration (years)** | NA | 5.3 ± 0.57  [<1–23] |
| **MDS-UPDRS total** | NA | 47.3 ± 3.1  [15–144]  n = 64 |
| **mH&Y** | NA | 2.3 ± 0.1  [0–4,5]  n = 64 |
| **PD-NMS** | NA | 9.8 ± 0.8  [0–24]  n = 62 |
| **MoCA** | NA | 25 ± 0.7  [1–30]  n = 64 |
| **LED (mg)** | NA | 609.4 ± 64.7  [0–2506]  n = 63 |

Data are presented as mean ± SEM, and values in squared brackets represent the range. CTR, controls; PD, Parkinson’s disease; MDS-UPDRS, Movement Disorders Society-Unified Parkinson’s Disease Rating Scale; mH&Y: modified Hoehn & Yahr stage; LED: Levodopa-equivalent dose. n: number of subjects with available data. Differences between the CTR and PD group were not significant.

**Supplemental Table S3: Differentially expressed miRNAs in the cerebrospinal fluid extracellular vesicles (Parkinson disease vs control comparison).**

| **miRNA** | **Mean of normalized counts** | **log2 (fold-change)** | **p-value** | **Adjusted p-value** |
| --- | --- | --- | --- | --- |
| *** hsa-miR-99a-5p** | 1194.6272 | -0.4457 | 1.000E-04 | 4.000E-03 |
| *** hsa-miR-501-3p** | 95.7068 | -0.6074 | 7.000E-04 | 1.530E-02 |
| hsa-miR-186-5p | 877.1926 | -0.1642 | 9.000E-04 | 1.700E-02 |
| hsa-miR-99b-5p | 3437.0253 | -0.4543 | 1.700E-03 | 2.790E-02 |
| *** hsa-miR-126-5p** | 46.25105 | 1.4468 | 8.500E-15 | 3.404E-12 |
| hsa-miR-126-3p | 9.3121 | 2.0096 | 1.260E-14 | 3.404E-12 |
| hsa-miR-138-5p | 5.2576 | 2.2042 | 1.740E-11 | 3.139E-09 |
| hsa-miR-9-5p | 139.3824 | 1.1141 | 1.530E-09 | 2.066E-07 |
| hsa-miR-219a-2-3p | 10.8027 | 2.0646 | 3.320E-09 | 3.584E-07 |
| hsa-miR-181b-5p | 135.139 | 0.7538 | 2.130E-08 | 1.916E-06 |
| hsa-miR-451a | 100.088 | 1.7425 | 6.920E-08 | 5.339E-06 |
| hsa-miR-181a-5p | 3161.1199 | 0.5621 | 8.400E-07 | 5.670E-05 |
| hsa-miR-486-5p | 564.2658 | 1.1222 | 1.870E-05 | 9.000E-04 |
| hsa-miR-184 | 5.8084 | 1.4741 | 2.990E-05 | 1.300E-03 |
| hsa-let-7b-5p | 347.1064 | 0.3857 | 6.180E-05 | 2.600E-03 |
| hsa-miR-98-5p | 74.9432 | 0.4504 | 8.860E-05 | 3.400E-03 |
| hsa-miR-127-3p | 304.7064 | 0.5361 | 2.950E-04 | 8.000E-03 |
| hsa-miR-144-3p | 11.6181 | 1.1301 | 1.311E-03 | 2.360E-02 |
| hsa-miR-769-5p | 65.5686 | 0.3278 | 1.759E-03 | 2.790E-02 |
| hsa-miR-211-5p | 6.4601 | 0.7716 | 1.968E-03 | 3.010E-02 |
| hsa-miR-129-5p | 5.7783 | 0.7821 | 2.032E-03 | 3.010E-02 |
| hsa-miR-16-5p | 554.9582 | 0.2504 | 2.160E-03 | 3.070E-02 |

MiRNAs found down-regulated and up-regulated in Parkinson’s disease (PD), sorted by the log2(fold-change). Candidates in bold represent the signature-miRNAs revealed by measure of relevance (MoR) and reliability analysis (RiA), followed by a machine-learning variable ranking method.[1, 2]

**Supplemental Table S4:** **Differentially expressed proteins in total cerebrospinal fluid (Parkinson disease vs control comparison).**

| **Protein (Entry name - UniProt)** | **q-value** | **log2 (Fold-change)** | **p-value** | **Adjusted p-value** |
| --- | --- | --- | --- | --- |
| VGF_HUMAN** | 4.998E-08 | -0.6148 | 2.293E-07 | 4.189E-05 |
| NEC1_HUMAN | 2.781E-07 | -0.5710 | 2.445E-06 | 2.680E-04 |
| NPY_HUMAN | 1.633E-07 | -0.4945 | 1.545E-03 | 2.566E-02 |
| CMGA_HUMAN* | 4.230E-08 | -0.4935 | 5.641E-05 | 3.091E-03 |
| SLIK1_HUMAN | 2.654E-07 | -0.4521 | 2.689E-04 | 7.855E-03 |
| CADM2_HUMAN | 1.570E-05 | -0.4410 | 7.839E-08 | 4.189E-05 |
| UBB;UBC;RS27A; RL40_HUMAN | 1.109E-07 | -0.4280 | 1.650E-05 | 1.292E-03 |
| VTM2A_HUMAN | 1.212E-07 | -0.4108 | 8.830E-05 | 4.032E-03 |
| PTPR2_HUMAN | 1.711E-07 | -0.4008 | 1.703E-07 | 4.189E-05 |
| SCG2_HUMAN* | 4.230E-08 | -0.3653 | 3.190E-06 | 2.914E-04 |
| APOC2_HUMAN* | 3.517E-07 | -0.3447 | 5.405E-03 | 4.778E-02 |
| 7B2_HUMAN | 5.992E-07 | -0.3258 | 4.169E-03 | 4.513E-02 |
| C99L2_HUMAN | 4.548E-08 | -0.3029 | 7.592E-05 | 3.782E-03 |
| CAD13_HUMAN | 5.837E-08 | -0.3009 | 1.826E-04 | 6.932E-03 |
| GFRA2_HUMAN | 2.174E-05 | -0.2994 | 2.087E-04 | 6.932E-03 |
| APLP1_HUMAN | 4.352E-08 | -0.2990 | 3.151E-04 | 8.634E-03 |
| AGRB2_HUMAN | 5.618E-08 | -0.2943 | 1.974E-04 | 6.932E-03 |
| TNR21_HUMAN | 7.257E-03 | -0.2832 | 1.242E-03 | 2.269E-02 |
| PRRT3_HUMAN | 2.914E-07 | -0.2826 | 1.664E-03 | 2.605E-02 |
| CSTN3_HUMAN | 1.465E-06 | -0.2825 | 3.885E-03 | 4.435E-02 |
| OPCM_HUMAN | 5.115E-08 | -0.2659 | 4.314E-03 | 4.513E-02 |
| CP089_HUMAN | 3.109E-07 | -0.2619 | 5.061E-03 | 4.757E-02 |
| GUAD_HUMAN | 7.434E-08 | -0.2457 | 4.365E-03 | 4.513E-02 |
| NRCAM_HUMAN | 3.823E-08 | -0.2413 | 6.049E-03 | 4.948E-02 |
| EPHA4_HUMAN** | 3.823E-08 | -0.2387 | 6.359E-04 | 1.429E-02 |
| PMGT1_HUMAN | 9.793E-08 | -0.2324 | 1.928E-05 | 1.321E-03 |
| TGON2_HUMAN | 8.357E-05 | -0.2302 | 9.793E-04 | 1.917E-02 |
| CGRE1_HUMAN | 4.564E-08 | -0.2260 | 1.420E-03 | 2.431E-02 |
| NPDC1_HUMAN | 1.755E-07 | -0.2260 | 5.011E-03 | 4.757E-02 |
| FAM3C_HUMAN | 4.230E-08 | -0.2104 | 3.992E-04 | 1.042E-02 |
| SPRL1_HUMAN | 5.236E-08 | -0.2099 | 2.428E-03 | 3.326E-02 |
| AP2B1_HUMAN | 1.182E-06 | -0.2084 | 4.604E-05 | 2.803E-03 |
| NECT1_HUMAN | 6.035E-07 | -0.2059 | 2.536E-03 | 3.339E-02 |
| NELL2_HUMAN | 4.352E-08 | -0.2049 | 4.113E-03 | 4.513E-02 |
| NBL1_HUMAN | 3.909E-07 | -0.1975 | 2.672E-03 | 3.405E-02 |
| PTPRF_HUMAN | 2.105E-07 | -0.1952 | 6.012E-04 | 1.429E-02 |
| PTPRS_HUMAN | 2.543E-07 | -0.1828 | 1.029E-03 | 1.945E-02 |
| CD166_HUMAN | 5.103E-08 | -0.1818 | 1.756E-03 | 2.673E-02 |
| C1R_HUMAN | 6.069E-08 | -0.1795 | 5.925E-03 | 4.948E-02 |
| T132A_HUMAN | 1.144E-07 | -0.1779 | 3.471E-03 | 4.047E-02 |
| AMD_HUMAN | 4.352E-08 | -0.1760 | 6.010E-03 | 4.948E-02 |
| PTPRD_HUMAN | 1.678E-07 | -0.1650 | 4.467E-03 | 4.533E-02 |
| GPX3_HUMAN | 4.410E-08 | -0.1595 | 5.280E-03 | 4.757E-02 |
| ALDOA_HUMAN | 4.410E-08 | -0.1326 | 5.983E-03 | 4.948E-02 |
| MMRN2_HUMAN | 1.086E-06 | -0.1308 | 2.865E-03 | 3.568E-02 |
| LTBP4_HUMAN | 1.700E-07 | 0.1890 | 6.519E-04 | 1.429E-02 |
| A1BG_HUMAN | 3.823E-08 | 0.2074 | 2.223E-03 | 3.124E-02 |
| LYSC_HUMAN | 7.022E-08 | 0.2103 | 5.172E-03 | 4.757E-02 |
| KIT_HUMAN | 7.923E-04 | 0.2126 | 1.376E-03 | 2.431E-02 |
| CBPQ_HUMAN | 7.310E-08 | 0.2175 | 4.459E-04 | 1.111E-02 |
| ZA2G_HUMAN | 4.352E-08 | 0.2213 | 6.877E-04 | 1.449E-02 |
| PGS2_HUMAN | 4.548E-08 | 0.2216 | 2.193E-03 | 3.124E-02 |
| FSTL5_HUMAN | 5.638E-05 | 0.2549 | 4.969E-03 | 4.757E-02 |
| KNG1_HUMAN* | 4.479E-08 | 0.2610 | 5.295E-03 | 4.757E-02 |
| RET4_HUMAN | 4.927E-08 | 0.2885 | 5.781E-03 | 4.948E-02 |
| DNS2A_HUMAN | 1.554E-04 | 0.3031 | 1.301E-04 | 5.483E-03 |
| APOA4_HUMAN* | 4.087E-08 | 0.3136 | 8.825E-04 | 1.791E-02 |
| IPSP_HUMAN | 3.823E-08 | 0.3156 | 2.723E-04 | 7.855E-03 |
| CBPB2_HUMAN | 4.230E-08 | 0.3261 | 4.745E-03 | 4.728E-02 |
| APLD1_HUMAN | 1.444E-07 | 0.3310 | 1.619E-06 | 2.218E-04 |
| LV147_HUMAN | 1.093E-06 | 0.3388 | 3.110E-03 | 3.705E-02 |
| KAIN_HUMAN | 4.230E-08 | 0.3443 | 1.607E-03 | 2.589E-02 |
| TM198_HUMAN | 1.043E-07 | 0.3723 | 4.268E-03 | 4.513E-02 |
| KLKB1_HUMAN | 1.922E-07 | 0.4094 | 3.046E-03 | 3.705E-02 |
| KV401_HUMAN | 2.927E-07 | 0.6094 | 2.150E-04 | 6.932E-03 |
| HV102_HUMAN | 2.209E-04 | 0.6113 | 2.100E-03 | 3.110E-02 |
| IGHG4_HUMAN | 5.500E-08 | 0.8424 | 2.559E-03 | 3.339E-02 |

Proteins found down-regulated and up-regulated in Parkinson’s disease (PD), sorted by the log2(fold-change). * Proteins already implicated/found deregulated in PD[3, 4][5][6, 7]. ** Proteins already postulated as PD biomarkers[8][9].

**REFERENCES**

1. Yassouridis A, Ludwig T, Steiger A, Leisch F (2012) A New Way of Identifying Biomarkers in Biomedical Basic-Research Studies. PLoS ONE 7:e35741. https://doi.org/10.1371/journal.pone.0035741

2. Denk J, Boelmans K, Siegismund C, et al (2015) MicroRNA Profiling of CSF Reveals Potential Biomarkers to Detect Alzheimer`s Disease. PLOS ONE 10:e0126423. https://doi.org/10.1371/journal.pone.0126423

3. Cocco C, D’Amato F, Noli B, et al (2010) Distribution of VGF peptides in the human cortex and their selective changes in Parkinson’s and Alzheimer’s diseases. Journal of Anatomy 217:683–693. https://doi.org/10.1111/j.1469-7580.2010.01309.x

4. Rüetschi U, Zetterberg H, Podust VN, et al (2005) Identification of CSF biomarkers for frontotemporal dementia using SELDI-TOF. Experimental neurology 196:273–281

5. Rotunno MS, Lane M, Zhang W, et al (2020) Cerebrospinal fluid proteomics implicates the granin family in Parkinson’s disease. Sci Rep 10:2479. https://doi.org/10.1038/s41598-020-59414-4

6. Gregório ML, Pinhel MAS, Sado CL, et al (2013) Impact of Genetic Variants of Apolipoprotein E on Lipid Profile in Patients with Parkinson’s Disease. BioMed Research International 2013:1–7. https://doi.org/10.1155/2013/641515

7. Boerger M, Funke S, Leha A, et al (2019) Proteomic analysis of tear fluid reveals disease-specific patterns in patients with Parkinson’s disease – A pilot study. Parkinsonism & Related Disorders 63:3–9. https://doi.org/10.1016/j.parkreldis.2019.03.001

8. Cocco C, Corda G, Lisci C, et al (2020) VGF peptides as novel biomarkers in Parkinson’s disease. Cell Tissue Res 379:93–107. https://doi.org/10.1007/s00441-019-03128-1

9. Shi M, Movius J, Dator R, et al (2015) Cerebrospinal Fluid Peptides as Potential Parkinson Disease Biomarkers: A Staged Pipeline for Discovery and Validation. Mol Cell Proteomics 14:544–555. https://doi.org/10.1074/mcp.M114.040576
