# Supplementary material for: MicroRNAs from extracellular vesicles as a signature for Parkinson's disease
Source: Clin Transl Med. 2021 Apr 5;11(4):e357. doi: 10.1002/ctm2.357 (PMC8021010; doi:10.1002/ctm2.357)
Supplement: Supplementary file 2 — Supporting information [file CTM2-11-e357-s002.docx]

SUPPORTING INFORMATION FOR

**MicroRNAs from extracellular vesicles as a signature for Parkinson’s disease**

Lucas Caldi Gomes*, Anna-Elisa Roser*, Gaurav Jain, Tonatiuh Pena-Centeno, Fabian Maass, Lukas Schilde, Caroline May, Anja Schneider, Mathias Bähr, Katrin Marcus, André Fischer, Paul Lingor

**METHODS**

**Participants**

CSF samples from patients with PD (n = 117) and control subjects (CTR; n = 105) were selected from the CSF Biobank of the Department of Neurology, University Medical Center, Göttingen (Tables 1, 2). All PD patients (diagnosed according to UK Brain Bank criteria, compliant with Movement Disorders Society criteria) were clinically examined, using the Movement Disorders Society—Unified Parkinson’s Disease Rating Scale (MDS-UPDRS), Parkinson’s disease non-motor scale (PD-NMS), modified Hoehn & Yahr scale (mH&Y), and the Montreal Cognitive Assessment (MoCA). CSF levels of Tau, phospho-Tau (pTau), and β-amyloid 40 and 42 were determined. Disease onset was defined as the time point when the first motor symptoms became apparent. In patients treated with anti-Parkinsonian medication, the levodopa-equivalent dose (LED) was calculated. Patients with known familial PD were excluded. Only patients without signs of neurodegenerative, neuroinflammatory, or acute ischemic central nervous system diseases were included as CTR.

Written consent was obtained from all patients or caregivers, and permission from the Ethics Committee of the University Medical Center Göttingen was obtained prior to the initiation of the study (No. 13/11/12). The study conformed to the Code of Ethics of the World Medical Association (Declaration of Helsinki).

**Lumbar Puncture and CSF Handling**

Lumbar punctures (LP) were performed between 9 a.m. and 2 p.m. to minimize the influence of circadian variability. CSF (6‒10 mL) was collected in polypropylene tubes and centrifuged at 2000 × *g* for 20 min (4°C) within 30 min after LP. The pellet was discarded, and the CSF was snap-frozen and stored at -80°C until vesicle isolation or protein lysate preparation. Samples were evaluated for erythrocyte numbers prior to storage, and only CSF samples with less than 100 erythrocytes per µL were used.

**RNA Isolation from Extracellular Vesicles in Human CSF**

Isolation of EVs was performed according to previously published protocols[1, 2] with the following adaptations: for each donor, 4.5 ± 0.5 mL CSF was thawed on ice. The CSF was diluted 1:2 with sterile cold PBS and ultracentrifuged at 110,000 × *g* for 3 h at 4°C (ACC/DCC 09/00) (Rotor: Sorvall TH-641). Total RNA was isolated from the pellet containing the CSF vesicular fraction using TRIzol (Invitrogen, Carlsbad, CA, USA). RNA quality and quantity were assessed by Bioanalyzer using the Agilent RNA 6000 Pico Assay kit (Agilent, Santa Clara, CA, USA) (Fig. 1B). For the characterization of the EVs isolated with this protocol, three human CSF pools of 4 ml each were evaluated by nanoparticle tracking analysis (NTA). The supernatants were collected and the EV pellets were then dissolved in 150 uL dPBS and vortexed for 2 mins. Both EV pellet and supernatant were measured using NanoSight NS500 instrument equipped with a 532 nm laser (NanoSight, Malvern Instruments Ltd, Malvern, UK) with the same settings, four times for 30 seconds each. Each video contained a minimum of 200 tracks. The obtained measurements were analyzed with the NanoSight Tracking Analysis 3.2 software.

**Small RNA Sequencing, Differential Expression Analyses, and Machine Learning**

The total amount of isolated RNA remaining after quality control (5 µL) was used for the preparation of small RNA libraries using the TruSeq Small RNA Library Prep Kit (Illumina, San Diego, CA, USA). Sequencing was performed on the Illumina HiSeq 4000, generating 50-bp single-end reads (10‒20 Mio reads/sample) (Illumina). Samples from Cohorts 1 and 2 were sequenced in completely independent runs using the exact same settings. Briefly, base calls in the per-cycle BCL files were converted to the per-read FASTQ format using Illumina’s bcl2fastq (v1.8.4). Base-calling, adapter trimming, and demultiplexing were performed, and the quality of the raw sequencing data was checked using FastQC (v0.11.5). Reads were first mapped to a database of documented miRNA sequences, followed by mapping to a reference genome, and the remaining unmapped reads were mapped to the human genome using Bowtie (v1.1.2). Differential expression analysis was performed to compare miRNA abundance between samples after removing linear shifts associated with batch effects with limma’s removeBatchEffect function and correction of unwanted sources of variation (RUVs) by RUVSeq (v1.8.0) using DESeq2 (v1.14.1). Heatmaps were created using a custom Python script for Python v.2.7.1 and Matplotlib 1.5.1.

To select the most important features for differentiation of PD and CTR samples, an iterative feature selection approach was used on the discovery cohort samples (cohort 1). First, Measure of Relevance (MoR)[3] and reliability analysis (RiA)[4] were performed in iteration 1. This was followed by machine learning variable ranking, to filter out low-ranking miRNAs from iteration 1[5]. For that, random forest was selected, since it is an out-of-the-box learning algorithm that provides both classification and predictive performance with relatively little hyperparameter tuning. By using out-of-bag (OOB) samples (left-out samples that were excluded from building the current tree in a particular iteration) to calculate the variable importance and to estimate the error in prediction, it serves as inherent cross-validation as well, providing a better estimation of errors and variable importance[6, 7]. We also used a 5-fold CV to avoid any overfitting.

The performance of the selected miRNAs as a PD signature was evaluated in the independent validation cohort (cohort 2), using the random forest algorithm in R (v3.2.2) with the random forest package (v4.6.14). Input parameters, such as the average error and average number of trees, were calculated by 10-fold cross-validation on the discovery cohort data. With the optimized input parameters, a model was trained with stratified sampling and class weights to minimize false-negatives.

To estimate the performance of the model in the independent validation cohort, receiver operating characteristic (ROC) curve analysis was used. The area under the curve (AUC) values, with smoothing, and the confidence interval for the AUC values were plotted using the pROC package with 500 stratified bootstrap iterations. Pairwise sample correlation heatmaps showing miRNA abundance changes between PD and CTR samples, as well as within the PD samples of the discovery cohort, were calculated using Bayesian hierarchical clustering.[8]

**Sample Preparation, Mass Spectrometry and Data Processing**

Samples of 64 PD and 61 CTR patients were used for proteomics analysis, of which 23 PD and 35 CTR samples overlapped with the miRNA discovery cohort 1 (Table 2). Protein lysates were prepared from 1 mL of each CSF sample, followed published protocols.[9, 10] After proteolytic digestion, the peptides were resuspended in 20 μL of 0.1 % (v/v) trifluoroacetic acid. Eight microliters of each sample were used to calculate the protein concentration by amino acid analysis.[11] Eventually, each sample was spiked with 1 µL of indexed retention time (iRT) peptides (Biognosys AG, Schlieren, Switzerland).

Peptide separation was performed as described previously [9, 10]. For the library generation, the Q Exactive^TM^ mass spectrometer (Thermo Fisher Scientific GmbH) was operated in data-dependent acquisition mode: total run time 135 min, range for full scans 400–1,400 m/z, full scan resolution 70,000, and MS/MS scans were recorded with a resolution of 35,000. HCD fragmentation of the 10 most common precursor ions was performed at a stepped normalized collisional energy (NCE) of 25.5, 27, and 30 (AGC 3e6, 20-ms maximum injection time).

For the sample analysis, the instrument was operated in data-independent acquisition mode: total run time 135 min, range for full scans of 400–1,400 m/z, full scan resolution 70,000, MS/MS scans divided into 19 overlapping windows with a width of 56 m/z and with a resolution of 35,000. HCD fragmentation was performed at a stepped NCE 25.5, 27, and 30 (AGC 3e6, 20-ms maximum injection time).

The analysis was performed using Spectronaut^TM^ Pulsar Software (Biognosys AG, Schlieren, Switzerland) and MaxQuant (Max-Planck-Institut für Biochemie, Martinsried, Germany).[12] Normalization was performed outside of Spectronaut^TM^ Pulsar; instead, a locally estimated scatterplot smoothing normalization was applied. A regression analysis was performed to counteract any batch effects. In order to identify peptides and proteins that differ in abundance between the groups, the quotient of the mean values was calculated separately for peptides and proteins. A paired two-sample t-test was performed to calculate the p-value. The calculated p-value was adjusted using the false-discovery rate (FDR).

**Functional Annotation**

Computational target gene prediction for the signature-miRNAs was performed using the miRWalk database version 3.0.[13] Functional annotation (to Gene Ontology [GO] terms and Kyoto Encyclopedia of Genes and Genomes [KEGG] pathways) of the miRNA predicted target genes and protein expression data were performed within the data mining environment provided by the WebGestalt Platform[14] using standard parameters (R package ‘Apcluster’[15] used for clustering gene sets when performing GO term-enrichment for biological processes), STRING Platform v.11[16] using default settings, and the DAVID 6.8 platform[17] using an Expression Analysis Systemic Explorer (EASE) score of 0.1 and a minimum number of 2 counts. Additionally, target genes already experimentally validated for these miRNAs were functionally annotated to KEGG pathways as described above, to verify the involvement of miRNAs in PD-related pathways further.

**Reverse Transcription and Quantitative Real-Time Polymerase Chain Reaction**

RNA isolation for the quantitative polymerase chain reaction (qPCR)-validation cohort (cohort 3) was performed with the addition of a synthetic cel-miR-39-3p spike-in (10 pM; 5'-UCACCGGGUGUAAAUCAGCUUG-3' with 5'-phosphorylation; Invitrogen, Carlsbad, CA, USA) as an exogenous control. Reverse transcription (RT) was performed using the TaqMan MicroRNA RT Kit (Applied Biosystems, Foster City, CA, USA). Custom RT and pre-amplification primer pools were prepared, including the TaqMan MicroRNA Assays for hsa-miR-126-3p, hsa-miR-126-5p, hsa-miR-186-5p, hsa-miR-451a, hsa-miR-99a-5p, hsa-miR-501-3p, hsa-miR-191-5p, and cel-miR-39-3p (RT- and PCR-primers, respectively; Applied Biosystems). Quantitative RT-PCR (qRT-PCR) was performed in 50 cycles on a QuantStudio3 system (Applied Biosystems) with TaqMan Universal Master Mix II (No AmpErase UNG; Applied Biosystems). Abundance was normalized to the geometric mean (GM_CTR_) of the Ct values for the exogenous control cel-miR-39-3p and the endogenous control hsa-miR-191-5p (the latter showed no difference in abundance for PD and CTR in sequencing and is recommended by the manufacturer as a control). The ΔCt (ΔCt = Ct_miRNA_–GM_CTR_) was calculated and relative expression levels (REL) were analyzed (REL = 2–^ΔCt^).

**Statistical Analyses**

Statistical analyses of demographical/clinical data and qRT-PCR results were performed using GraphPad Prism 8. For qRT-PCR experiments, data are shown as mean ± SEM. Differences between PD and CTR groups were analyzed by unpaired *t*-tests (two-sided). Between-group comparisons of the demographical/clinical data were performed by one-way analysis of variance. Differences were considered significant at p < 0.05.

**SUPPLEMENTAL REFERENCES**

1. Akers JC, Ramakrishnan V, Yang I, et al (2016) Optimizing preservation of extracellular vesicular miRNAs derived from clinical cerebrospinal fluid. CBM 17:125–132. https://doi.org/10.3233/CBM-160609

2. Jain G, Stuendl A, Rao P, et al (2019) A combined miRNA–piRNA signature to detect Alzheimer’s disease. Transl Psychiatry 9:250. https://doi.org/10.1038/s41398-019-0579-2

3. Yassouridis A, Ludwig T, Steiger A, Leisch F (2012) A New Way of Identifying Biomarkers in Biomedical Basic-Research Studies. PLoS ONE 7:e35741. https://doi.org/10.1371/journal.pone.0035741

4. Denk J, Boelmans K, Siegismund C, et al (2015) MicroRNA Profiling of CSF Reveals Potential Biomarkers to Detect Alzheimer`s Disease. PLOS ONE 10:e0126423. https://doi.org/10.1371/journal.pone.0126423

5. Guyon I, Elisseeff A (2003) An Introduction to Variable and Feature Selection. Journal of Machine Learning Research 3:1157–1182

6. Díaz-Uriarte R, Alvarez de Andrés S (2006) Gene selection and classification of microarray data using random forest. BMC Bioinformatics 7:3. https://doi.org/10.1186/1471-2105-7-3

7. Genuer R, Poggi J-M, Tuleau C (2008) Random Forests: some methodological insights. arXiv:08113619 [stat]

8. Savage R, Cooke E, Darkins R XY (2019) BHC: Bayesian Hierarchical Clustering. R package version 1.36.0.

9. Barkovits K, Linden A, Galozzi S, et al (2018) Characterization of Cerebrospinal Fluid via Data-Independent Acquisition Mass Spectrometry. J Proteome Res 17:3418–3430. https://doi.org/10.1021/acs.jproteome.8b00308

10. Schilde LM, Kösters S, Steinbach S, et al (2018) Protein variability in cerebrospinal fluid and its possible implications for neurological protein biomarker research. PLoS ONE 13:e0206478. https://doi.org/10.1371/journal.pone.0206478

11. Guntermann A, Steinbach S, Serschnitzki B, et al (2019) Human tear fluid proteome dataset for usage as a spectral library and for protein modeling. Data in Brief 23:103742. https://doi.org/10.1016/j.dib.2019.103742

12. Cox J, Mann M (2008) MaxQuant enables high peptide identification rates, individualized p.p.b.-range mass accuracies and proteome-wide protein quantification. Nat Biotechnol 26:1367–1372. https://doi.org/10.1038/nbt.1511

13. Sticht C, De La Torre C, Parveen A, Gretz N (2018) miRWalk: An online resource for prediction of microRNA binding sites. PLoS ONE 13:e0206239. https://doi.org/10.1371/journal.pone.0206239

14. Liao Y, Wang J, Jaehnig EJ, et al (2019) WebGestalt 2019: gene set analysis toolkit with revamped UIs and APIs. Nucleic Acids Research 47:W199–W205. https://doi.org/10.1093/nar/gkz401

15. Bodenhofer U, Kothmeier A, Hochreiter S (2011) APCluster: an R package for affinity propagation clustering. Bioinformatics 27:2463–2464. https://doi.org/10.1093/bioinformatics/btr406

16. Szklarczyk D, Gable AL, Lyon D, et al (2019) STRING v11: protein–protein association networks with increased coverage, supporting functional discovery in genome-wide experimental datasets. Nucleic Acids Research 47:D607–D613. https://doi.org/10.1093/nar/gky1131

17. Huang DW, Lempicki RA, Sherman BT (2009) Systematic and integrative analysis of large gene lists using DAVID bioinformatics resources. Nature Protocols 4:44–57. https://doi.org/10.1038/nprot.2008.211
